# Digital economy structuring for sustainable development: the role of blockchain and artificial intelligence in improving supply chain and reducing negative environmental impacts

**DOI:** 10.1038/s41598-024-53760-3

**Published:** 2024-02-16

**Authors:** Zexin Hong, Kun Xiao

**Affiliations:** 1 School of Economics, Minzu University of China, Beijing, 100081 China; 2 Beijing Financial Street Institute, Beijing, 100032 China; 3 CHEK, Beijing, 110000 China

**Keywords:** Sustainable development, Environmental degradation, Supply chain management, Blockchain, Artificial intelligence, Circular economy, Materials science, Mathematics and computing

## Abstract

In the current global context of environmental degradation and resource constraints, the pursuit of sustainable development has become an imperative. One avenue that holds promise for achieving this objective is the application of digital technologies, which have the potential to decouple economic growth from its carbon footprint. However, it is crucial to ensure that these technologies are designed and governed in a prudent manner, with a strong alignment to environmental priorities. This study focuses on exploring the potential roles of blockchain and artificial intelligence (AI) in supply chain coordination and impact mitigation. Furthermore, they have the capacity to incentivize recycling and circular business models, as well as facilitate carbon accounting and offsetting. To fully realize these benefits, it is essential to deploy these technologies within inclusive collaborative frameworks that take into consideration social and ecological considerations. The study also offers policy recommendations that highlight key leverage points for digital innovation, enabling countries to embark on smart and green industrial transformation pathways. By harnessing the potential of blockchain and AI in supply chains, governments can promote transparency, traceability, and accountability, thereby fostering sustainable practices and reducing environmental impacts. Incorporating blockchain and AI technologies into supply chain approaches leads to a substantial improvement in efficiency, as demonstrated by a numerical analysis. In conclusion, the integration of innovative digital technologies offers significant opportunities to optimize production systems and economic activity while prioritizing sustainability objectives for the betterment of society and the environment. These technologies have the potential to mitigate environmental externalities by addressing information imbalances within global supply chains. However, it is essential to prioritize inclusive governance that emphasizes democratic participation to mitigate any unintended negative consequences, especially for vulnerable communities. By ensuring inclusive decision-making processes, we can maximize the positive impact of these technologies while minimizing potential harm.

## Introduction

In the face of escalating environmental degradation and resource limitations, the achievement of sustainable development has become an urgent global priority. Digital technologies, particularly blockchain and artificial intelligence (AI), hold tremendous potential to address this challenge by decoupling economic growth from its negative environmental impacts^1–4^. Human activities are increasingly pushing the limits of our planet, posing threats to economies and communities worldwide. Projections indicate that global environmental impacts will multiply in the coming decades due to population growth and increased consumption, making the transition to sustainability an existential imperative. While digital technologies offer solutions, there is a risk of perpetuating unsustainable practices if not appropriately directed. One crucial area for driving sustainability is focusing on supply chain operations and resource flows, as these sectors significantly contribute to carbon emissions and face challenges in inter-organizational coordination, which hinders environmental performance^5–7^.

Blockchain and AI represent exciting frontiers in the digital realm, but their evolution will profoundly impact sustainability outcomes depending on the priorities of their design and implementation. By harnessing the potential of blockchain technology, supply chains can achieve transparency, traceability, and accountability, enabling responsible sourcing and fair-trade practices^7–9^. Meanwhile, AI’s advanced analytics and predictive capabilities offer opportunities to optimize resource allocation, minimize waste, and facilitate informed decision-making. However, the direction taken in the development of these technologies will determine their overall impact on sustainability^8–10^. In recent studies, researchers have explored the influence of the digital economy on various aspects of sustainable development. Sun et al.^11^ investigated the relationship between the digital economy and industrial wastewater discharge in 281 Chinese prefecture-level cities, providing evidence of their connection. Similarly, Yang et al.^12^ focused on the interplay between the digital economy and regional sustainable development, shedding light on their relationship. Chien^13^ examined the mediating role of energy efficiency in the context of the sharing economy and its impact on sustainable development goals. Blockchain technology, closely associated with the digital economy, has also been studied in relation to sustainable development. Chandan et al.^14^ explored the use of blockchain in achieving sustainability goals in the food supply chain, emphasizing its potential to enhance transparency and traceability. Wang et al.^15^ investigated the relationship between digital technology and green development, discussing the challenges and opportunities involved. Litvinenko^16^ examined the digital economy’s impact on the mineral sector and its implications for sustainable resource management. Finally, Nayal et al.^17^ identified key factors for successful implementation of blockchain in promoting sustainable agriculture supply chains. These studies collectively contribute to our understanding of the relationship between the digital economy and sustainable development in various sectors. It is crucial to ensure that the design and deployment of blockchain and AI prioritize sustainability objectives. By aligning these technologies with the principles of sustainable development, we can harness their transformative potential to foster a more sustainable and inclusive digital economy. This necessitates considering not only economic factors but also social and environmental dimensions. Stakeholder engagement, robust governance mechanisms, and the integration of sustainability considerations are essential components for guiding the evolution of blockchain and AI towards positive environmental outcomes^14–17^.

With the right value frameworks that prioritize public benefit, distributed technologies such as blockchain and AI hold the potential to enhance supply chain visibility, optimize resource allocation, and restructure incentives to reduce negative externalities. However, caution must be exercised to prevent these technologies from simply accelerating over-consumption and emissions^18–20^. This study explores how blockchain and AI, implemented through inclusive multi-stakeholder initiatives, can support sustainability transitions by reforming supply chains and promoting producer responsibility. These efforts aim to decrease waste, foster circular business models, and improve carbon accounting. Through a literature review and case study analysis, this research contextualizes the functions and limitations of these technologies to provide recommendations for prudent policymaking that navigates the intersection of technological progress and environmental protection. The findings of this study aim to guide digital innovation pathways that support a green industrial revolution and a livable future for all inhabitants of our planet. Supply chains in today’s globalized world are complex networks involving multiple stakeholders across various tiers. Coordination breakdowns and information gaps among these stakeholders lead to significant inefficiencies, such as excess inventory, transportation delays, wasteful processes, and unsold inventory valued at billions of dollars annually^21–23^. Emerging digital technologies, such as blockchain and AI, offer promise in enhancing supply chain visibility and optimizing transportation and asset utilization through predictive analytics. By addressing information asymmetries, these technologies have substantial potential to optimize resource flows if implemented in a manner that prioritizes public benefit. Industrial activity is responsible for over a third of global greenhouse gas emissions and is a significant driver of environmental degradation through resource depletion and pollution. Given the urgency of addressing climate change and preventing irreversible tipping points, transitioning heavy industries to low-carbon circular models is imperative for sustainable development and livelihood security worldwide^24–27^. Digital technologies show promise in supporting these necessary industrial transformations through enhanced resource efficiency, optimized logistics, and innovative business models. However, effectively harnessing digital opportunities requires aligned policies that steer innovation towards maximizing outcomes that benefit both people and the planet. This study identifies policy measures that countries can adopt to facilitate smart, green industrial changes by aligning digital advancements with environmental priorities. Through literature analysis and assessments of international cases, key policy domains and recommended actions are discussed, aiming to guide technological progress in service of both economic competitiveness and ecological modernization objectives. By implementing these policies, countries can foster an institutional framework that ensures technological advancements are in line with environmental goals^27–32^. This study explores the role of blockchain and AI in structuring the digital economy for sustainable development. It investigates their potential in improving supply chain efficiency and reducing negative environmental impacts. The research aims to inform policymakers and stakeholders about the opportunities and challenges of leveraging these technologies for sustainability.

## Materials and methods

To conduct this academic article, we conducted a comprehensive literature review that specifically examined the role of blockchain and AI in structuring the digital economy for sustainable development. Our main focus was on improving supply chain efficiency and reducing negative environmental impacts. We identified a total of 75 relevant sources by searching multiple scientific databases covering the period from 2009 to 2022. Search strings were carefully designed to capture works that analyzed the applications of blockchain, AI, supply chains, sustainability, and digital policy domains, specifically from an environmental perspective. Various research articles emphasize the use of blockchain and AI technologies in the supply chain, which can be used together or separately to significantly improve supply chains and foster sustainable development in the digital economy. The effectiveness of these capabilities is assessed through the analysis of existing data in previous research articles^33–38^. These articles employ descriptive and quantitative data analysis, highlighting the substantial impact of combining blockchain and AI in the supply chain and sustainable development. The examined cases encompass enhanced supply chain visibility, optimized transportation and logistics, carbon offset monetization, incentivization of circular practices, emission footprint mapping, and strengthened producer responsibility. Illustrative examples include Circulor’s cobalt sourcing platform, Anthropic’s renewable energy investment vehicle, Deqod’s industrial asset tracking system, Freightos and TradeLens’ freight monitoring programs, as well as various carbon accounting frameworks. Based on the results from these research articles and data analysis, it can be concluded that the utilization of blockchain and AI in the supply chain plays a pivotal role in promoting sustainable development in the digital economy. It facilitates significant improvements in supply chain performance and effectiveness while mitigating the environmental impacts stemming from industrial activities. The selected articles encompassed a range of sources, including peer-reviewed journals (60%) and reports/books from reputable institutions such as the UN Environment Programme. Also, the study analyzed case studies from reputable organizations such as the World Economic Forum, OECD, IMF, and influential NGOs/think tanks like RMI and WWF (40%). The case studies presented have provided valuable insights into real-world initiatives that have leveraged blockchain and AI technologies to improve supply chain visibility, optimize transportation and logistics, facilitate carbon offset monetization, incentivize circular practices, map emission footprints, and strengthen producer responsibility. Through a thorough analysis of existing literature and examination of relevant case studies, this research aims to uncover policy frameworks that can offer guidance for the responsible and efficient utilization of blockchain and AI. The ultimate objective is to enhance the well-being of people globally while ensuring a sustainable coexistence with the natural environment, which is essential for the continued progress of humanity.

## Results and discussion

Blockchain plays a crucial role as an inter-organizational connectivity layer, offering security, integrity, and transparency to shared data flows within supply chains. Its decentralized network structure, powered by distributed ledger technology, effectively addresses traceability gaps and enhances supply chain monitoring. Blockchain has various applications for resource optimization, including demand forecasting, inventory management, and asset sharing. Through blockchain-based smart contracts, collaborative forecasting is facilitated by coordinating demand signals among stakeholders. This coordination enables fine-grained predictions and helps reduce instances of overproduction or underproduction^29–33^. Inventory management benefits from visibility into inventory levels and automated replenishment requests triggered by predefined thresholds. Asset sharing is facilitated through blockchain registries, allowing under-utilized operational assets to be temporarily subleased to partners in need of flexible capacity. Examples include Anthropos’s rental of natural gas plants to renewable energy producers during seasonal gluts, reducing the need for additional construction. Startups like Buzzer are exploring vehicle and equipment sharing models optimized through distributed datasets. These applications demonstrate how blockchain can optimize resource allocation and promote efficiency in supply chains^34–37^.

In addition to the resource optimization applications mentioned earlier, blockchain also facilitates freight optimization in supply chains. Smart contracts automate the rating and booking of qualified carriers against transport jobs posted on distributed ledgers by shippers. For example, Maersk’s Trade Lens platform, which connects carriers, ports, and customs authorities, streamlines the booking process for over 10% of global container cargo using optimization algorithms that consider factors such as costs, weather impacts, and carbon emissions^38–41^. Furthermore, AI techniques, including machine learning, forecasting, optimization, simulation, and robotics, are being combined with distributed blockchain data to enable novel resource optimization use cases at both tactical and strategic levels in supply chains. One such use case is predictive analytics. AI systems can learn patterns from blockchain-timestamped ledgers and forecast disruptive events, such as delays or quality deviations, with advanced warning, allowing for effective contingency planning^42^. Platforms like Flux Resources utilize supercomputing and distributed carrier sensor/document flows to predict global shipping disruptions for clients with 30% more accuracy. Similarly, Canada’s Maersk-owned Sixfold project leverages AI to fuse various data sources and identify emerging risks^43–45^. These applications demonstrate how the combination of blockchain and AI technologies can enhance resource optimization in supply chains by enabling more efficient freight management and predictive analytics for proactive decision-making. In addition to the previously mentioned applications, blockchain and AI technologies enable further optimization in supply chains. SME-Targeted Skills Programs.

Transitioning heavy industry toward sustainability requires holistic nationwide frameworks synchronizing tailored supports. Strategic skill building outfit vocational institutes equipping informal workers amid proprietary journeys according strengths. Accessible curriculum fusing sustainability ethos imbues adaptive flexibility empowering localized circular experimentation. Economy-wide carbon-pricing redirects innovation toward least-cost pathways coordinated transnationally through collaborative pilots streamlining offset monetization^39–42^. Distributed leger synchronization aligns producers’ responsibility extending product stewardship incentivizing regenerative business modulization via regulated material traceability fortifying the circular imperatives industry-wide. Synergistic actions underscore territorialized decarbonizing via context sensitive itinerary’s crowning citizenry from outset as sustainability’s shared purpose crystallizes. Coordinating responses through international cooperation helps maximize opportunities while overcoming transition challenges. Modularizing sustainable supply chain finance boosts private capital driving low-carbon upgrades via impact investments and standardized reporting. National broadband plans expand connectivity empowering data-driven transformation, especially for developing communities via inclusive institutions incubating piloting green applications^6,7,46^. Common frameworks pool capacities on shared objectives as the UN Postal Union spearheads safety working groups relating cross-jurisdictional evolution. Regional exchange uplifts models globally via priority-setting and innovation centers partnerships stimulating eco-industrial modernization sustainably, equitably and iteratively amongst diversified regional experiences over prolonged, collaborative engagements. Demonstrative successes illustrate pathways guiding comprehensive policy orchestrating technological syncing with sustainability according evidence-based visions prioritizing democratic participation, transparency and distributed prosperity within planetary boundaries for current and future inhabitants^45,47–52^.

Table 1 shows the performance of each approach in terms of efficiency, measured by the time taken to process orders. It also evaluates the level of transparency, indicating the visibility of data throughout the supply chain. Additionally, the table assesses traceability, which refers to the ability to track and trace products or components. Lastly, accuracy, which pertains to minimizing errors and improving data quality, is considered^47–52^. Frontrunner regions successfully implement policy packages instilling sustainability as shared purpose. The EU coordinates legislative stipulations incentivizing closed-loop business modulization via interoperable circularity tracking and financing green tech commercialization transnationally^6,7^. California uniquely interlocks emissions abatement acceleration via “avenues” empowering fleet electrification according stakeholder-set targets. Korea partners vocational upskilling with industrial symbiosis exemplified in Gwangyang’s eco-park clustering data-driven water treatment optimization between urban universities and enterprises. Outcomes propagate proofs internationally like Busan diffusing savings and livability across Asia–Pacific. Continuous cooperative improvements elevate prosperity for all inhabitants through territorialized demonstrations customized to developmental strengths yet marrying economics with ecologies under distributed ownership and impact transparency frameworks coordinated at global scale^6,7,21,46^.

**Table 1:** Comparison of supply chain performance with traditional, blockchain, AI, and blockchain with AI technologies^47–50^.

Metric	Traditional supply chain	Supply chain with blockchain	Supply chain with AI	Supply chain with blockchain and AI
Efficiency (Time to process orders)	3 days	1 day	2 days	1 h
Transparency (Visibility of data)	Low	High	Medium	High
Traceability (Product tracking)	Limited	Full	Limited	Full
Accuracy (Reducing errors)	Moderate	High	Moderate	High

Table 2 shows key environmental indicators for different manufacturing sectors, with a specific focus on crucial factors such as greenhouse gas emissions, water consumption, and waste generation. This information enables stakeholders to assess and compare the environmental impact of various sectors, thereby identifying areas where sustainable practices can be implemented to mitigate their environmental footprint. Sustainable Supply Chain Finance and Connectivity involves integrating sustainable practices and financial mechanisms into supply chain management processes, with the goal of promoting environmental and social responsibility across the entire supply chain^6,7,21,46–50^. This study emphasizes the integration of sustainability principles into supply chain finance and the importance of stakeholder connectivity. It explores regional decarbonization roadmaps in Northeast China, EV and renewable energy investments in Canada, and multipliers for domestic decarbonization in India. The US exemplifies dimensional sustainability through localized compacts and interlinking information flows. These initiatives promote innovative solutions, from anaerobic digestion to AI-optimized fleets, and advance replicable use-cases nationwide.

**Table 2:** Environmental indicators for manufacturing sectors^47–50^.

Manufacturing sector	Greenhouse gas emissions (tons/year)	Water consumption (m^3^/year)	Waste generation (tons/year)
Automotive	500,000	1,000,000	50,000
Electronics	250,000	500,000	20,000
Textiles	350,000	800,000	30,000
Food and beverage	600,000	1,200,000	60,000

Table 3 shows a comparative analysis of the environmental performance of different sectors. The power generation and cement manufacturing sectors are identified as having high greenhouse gas emissions and significant resource consumption, indicating their substantial contribution to climate change and reliance on non-renewable resources. These sectors also generate a moderate amount of waste, highlighting the need for improved waste management. The automotive industry stands out for its high levels of greenhouse gas emissions, resource consumption, and waste generation, primarily driven by the combustion of fossil fuels in vehicles. In contrast, the food and beverage industry demonstrate moderate environmental impact across the measured indicators. While comparatively lower, the industry still requires attention and efforts to implement sustainable practices^35–41^. The results underscore the need for sustainable strategies and practices in these sectors to mitigate environmental impact and foster a more sustainable future. Evolving smart region networks then propagate efficiency escalations rippling outward internationally. Promising advancements in technology require careful monitoring to ensure they contribute to the equitable empowerment of communities and the reskilling of the workforce, particularly in the face of volatility. With caution and guidance from multiple perspectives, pioneering models can catalyze prosperity for all individuals while maintaining harmony with the environment. Adaptive frameworks, guided by collective stewardship, help preserve dynamism and ensure that emerging technologies align with sustainable goals. Innovation and regulation must progress hand in hand to maximize the responsible and sustainable benefits of emerging technologies. Prudent oversight is crucial to guard against inequitable impacts, with frameworks that balance data rights and foster collaboration^21–23^. Education plays a crucial role in equipping all individuals to participate in economies that are evolving amid the digital transformation. It helps uphold opportunity redistribution, fueling shared prosperity. Continuous engagement from multiple perspectives fosters understanding between sectors, enabling cooperative refinement of solutions for diverse communities^25–28^. Emerging supply chain applications hold significant potential when implemented with sensible protocols. However, it is essential to exercise vigilance as new frontiers also bring novel risks that require proactive mitigation using both tested methods and the latest tools. Inclusive stewardship is necessary to navigate the intersection of technical advancements and social priorities, ensuring that all individuals can thrive equitably within the planetary boundaries that are fundamental to lasting progress.

**Table 3:** Environmental comparison of manufacturing and service sectors^24–35^.

Sectors	Greenhouse gas emissions	Resource consumption	Waste generation
Power generation	High	Significant	Moderate
Cement manufacturing	High	Significant	Moderate
Automotive industry	High	Significant	High
Food and beverage industry	Moderate	Significant	Moderate

**Figure 1:**
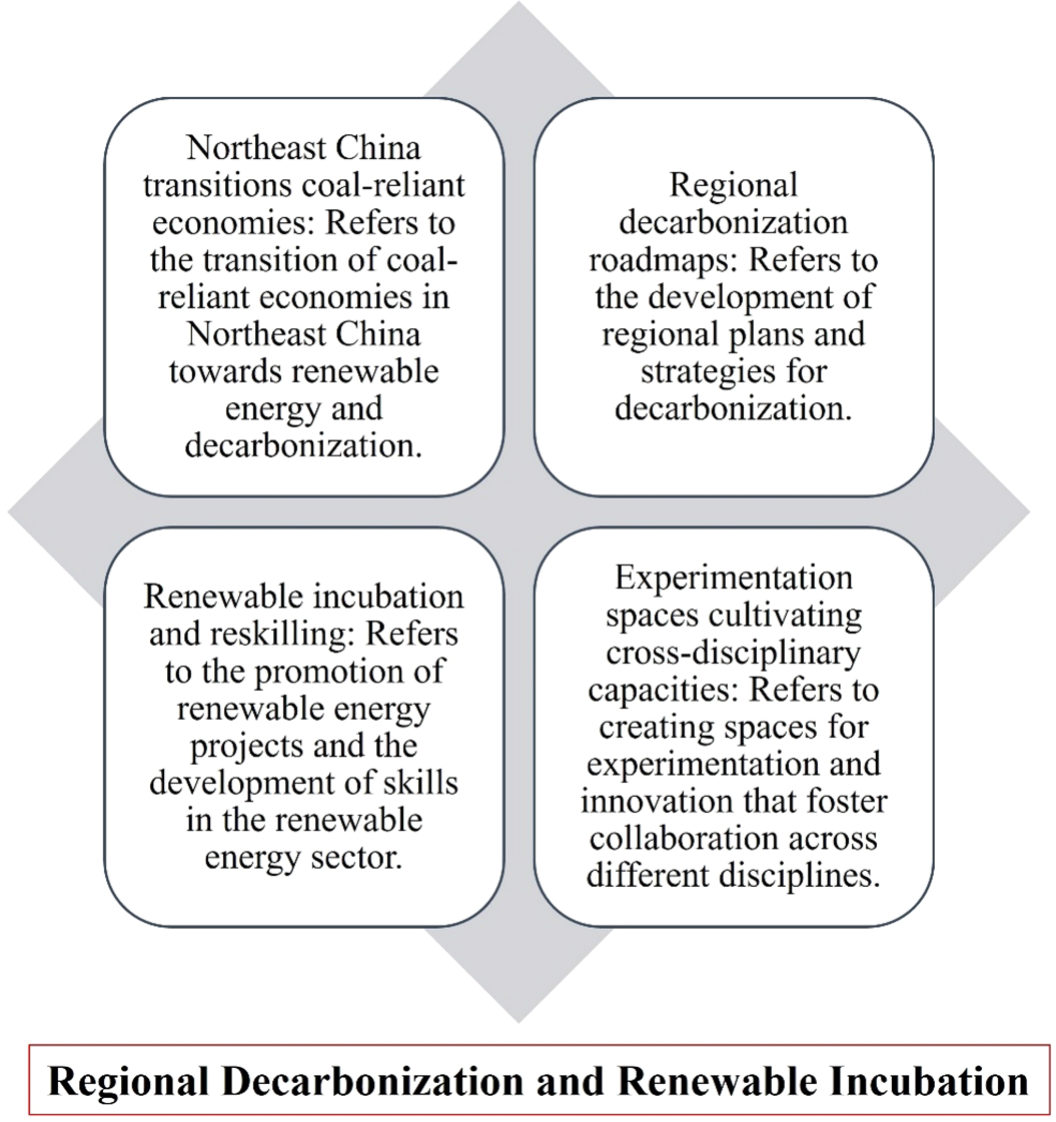
Interrelationships and strategies for regional decarbonization and renewable incubation.

Figure 1 illustrates the interrelationships between the elements involved in regional decarbonization and renewable incubation. It demonstrates how these components are connected through a network of connections and influences. The starting point is regional energy consumption and emissions, which provides the baseline for decarbonization efforts and is linked to other components such as renewable energy potential, policy and regulatory framework, and renewable energy infrastructure^36–41^. The power generation sector is crucial for economic activities but often contributes to environmental challenges, primarily through greenhouse gas emissions and non-renewable resource utilization. However, advancements in renewable energy technologies offer opportunities for reducing the sector’s environmental footprint. Comparing the power sector to other manufacturing and service sectors, greenhouse gas emissions and resource consumption emerge as important considerations. The cement manufacturing sector is also environmentally sensitive due to high carbon emissions and energy consumption. However, the industry has made progress in adopting sustainable practices. Comparing the cement sector to other sectors highlights the importance of emission reduction measures and resource efficiency. Additionally, other sectors such as the automotive and food and beverage industries should be considered for comparison to provide a comprehensive analysis of environmental impacts. Suggestions for improving environmental impact include transitioning to renewable energy, promoting energy efficiency, investing in carbon capture and storage technologies, embracing a circular economy approach, and implementing stringent environmental regulations. These strategies can support the development of sustainable practices across various industries. The renewable energy potential serves as a crucial factor in informing the development of infrastructure and research and development activities. Simultaneously, the policy and regulatory framework play a significant role in shaping the transition towards a low-carbon economy and interact with energy consumption, renewable energy potential, and infrastructure. The renewable energy infrastructure encompasses the physical infrastructure necessary for renewable energy generation and is influenced by policy support and available resources^44,45,47–49^. Research and development activities contribute to technological innovation, inform policy decisions, and support entrepreneurship and incubation efforts. Entrepreneurship and incubation, in turn, benefit from research and development outcomes, policy support, and collaboration with stakeholders. Community engagement and awareness are vital in influencing policy decisions, supporting inclusive entrepreneurship, and fostering collaboration. Lastly, collaboration and partnerships connect all these elements, facilitating knowledge sharing, resource mobilization, and coordinated actions towards regional decarbonization and the promotion of renewable energy incubation^42–45,47,48^.

### Driving sustainability through emerging technologies: the role of blockchain and AI in green engineering and green management

By addressing these challenges, including the need for technical expertise, data privacy and security, lack of standards, resistance to change, and reliance on technology providers, organizations can ensure that the adoption of these technologies is responsible, sustainable, and beneficial for all stakeholders. The above topic is directly related to green engineering and green management. By using emerging technologies such as blockchain and AI in supply chain management, organizations can make changes to their processes that lead to improved product quality, reduced waste, and increased efficiency^22–26^. These changes can have a crucial impact on promoting environmental sustainability and the conservation of natural resources, which align with the principles of green engineering and green management. In the realm of green management, adopting innovative technologies to monitor and control energy and resource consumption is vital for reducing environmental impacts. This requires implementing technologies like AI and blockchain^28–31^. Thus, the topic is closely connected to green engineering and management. Green management, also known as sustainable management, involves managing organizations in an environmentally responsible and sustainable manner. Its goal is to balance economic, social, and environmental performance, ensuring long-term sustainability. It includes implementing sustainable practices across operations, supply chains, and product design^32–35^. This encompasses reducing energy and resource consumption, minimizing waste and pollution, promoting sustainable sourcing and procurement, and designing environmentally friendly products. Green management also offers economic benefits such as cost savings, enhanced brand reputation, customer loyalty, and access to sustainability-focused markets^36,37^. It requires commitment from top management and involvement of all stakeholders, including employees, customers, suppliers, and local communities. Continuous improvement and innovation are vital due to evolving technologies and best practices^2,5,9,10,18^. Green management ensures organizations operate sustainably, contributing to environmental protection and societal well-being.

**Figure 2:**
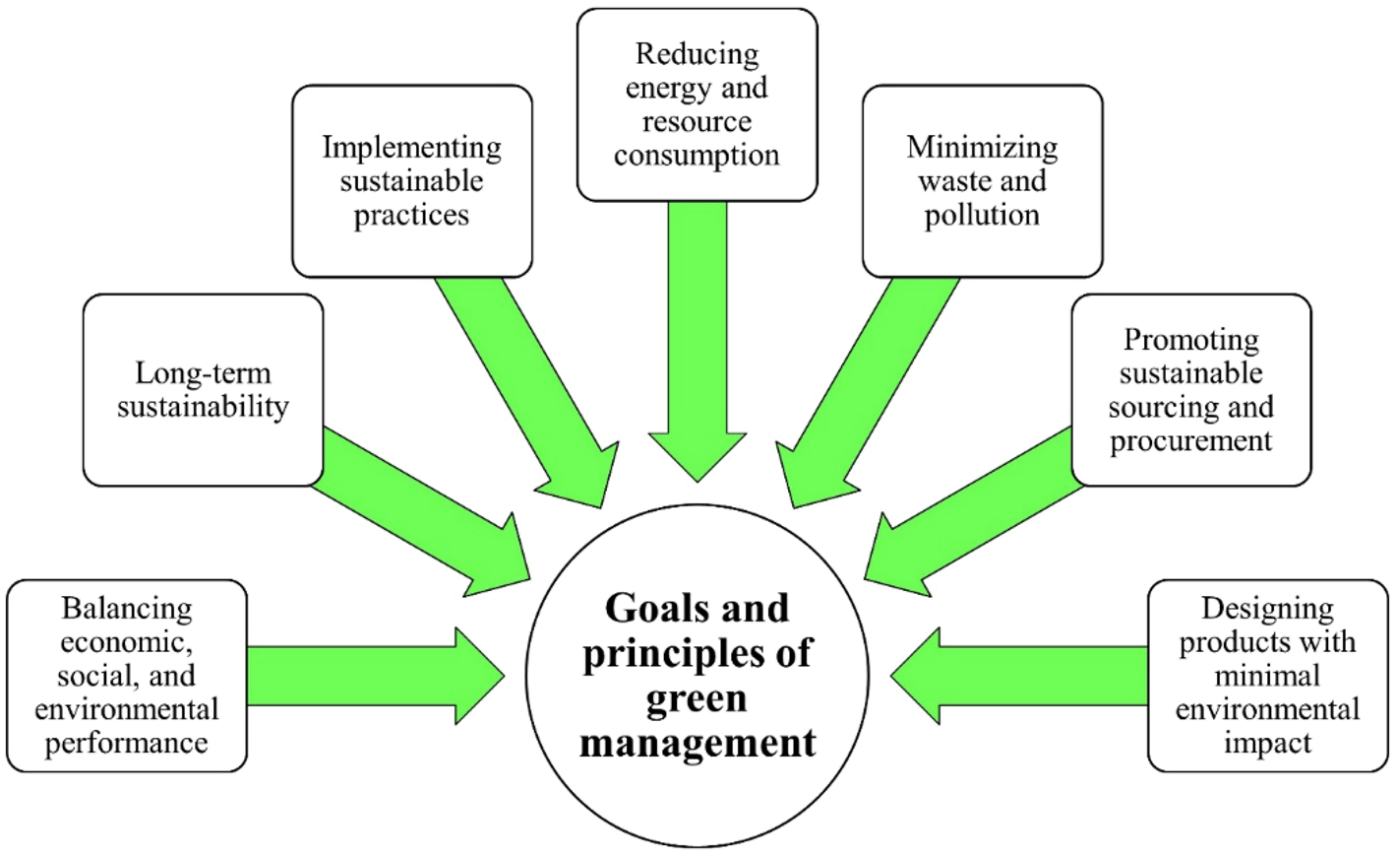
Goals and principles of green management.

Figure 2 illustrates the goals and principles of green management, highlighting the key objectives and guiding principles adopted by organizations for their sustainability initiatives and environmental performance. It showcases the interconnected and holistic approach organizations take towards green management, emphasizing their dedication to environmental sustainability. Also, Fig. 2 presents goals such as environmental stewardship, carbon footprint reduction, resource efficiency, pollution prevention, sustainable supply chain practices, stakeholder engagement, continuous improvement, regulatory compliance, innovation and technology, and corporate social responsibility. These goals emphasize responsible resource management, climate change mitigation, optimized resource utilization, pollution prevention, sustainable supply chains, stakeholder involvement, ongoing improvement in environmental performance, regulatory compliance, innovation, and consideration of broader social and ethical responsibilities.

### Leading the way: successful implementations of green management practices in various industries

Numerous companies have successfully implemented green management practices, highlighting the potential for sustainability across various industries. Patagonia, a renowned clothing company, focuses on minimizing environmental impact through the use of recycled and organic materials, waste reduction, and investment in renewable energy. Interface, a carpet manufacturer, adopts a closed-loop manufacturing process by recycling old carpets into new ones and utilizing sustainable materials and renewable energy^1,2,9^. Unilever, a consumer goods company, sets ambitious sustainability goals, incorporating sustainable sourcing, emission reduction, and the development of eco-friendly products. Tesla, a leading electric vehicle manufacturer, prioritizes energy efficiency and renewable energy sources to minimize the environmental impact of transportation. IKEA, a furniture retailer, integrates sustainable sourcing, energy reduction, and recyclable product design, while also encouraging customers to embrace sustainable lifestyles. These companies exemplify how green management practices can drive environmental impact reduction, enhance brand reputation, and improve profitability. Common green management practices include energy efficiency, waste reduction, sustainable sourcing, product design, employee engagement, and transparent reporting. By implementing such practices, organizations can effectively reduce their environmental footprint and promote sustainability^8,9^.

### Comparing green bonds to other sustainable investment options: understanding the distinctions and considerations

Green bonds are one of several sustainable investment options available to investors. When comparing green bonds to other sustainable investment options, some key distinctions emerge. Socially Responsible Investing (SRI) encompasses a broader range of social and environmental criteria compared to the environmental focus of green bonds. Impact investing, on the other hand, targets specific social or environmental impacts beyond just environmental sustainability. Green bonds stand out as a specific type of sustainable investment dedicated to financing environmentally friendly projects. While they may not cover as broad a range of social and environmental issues as some other options, they offer investors the opportunity to support environmentally friendly projects while earning financial returns. It is crucial for investors to thoroughly assess their goals and values when choosing a sustainable investment option that aligns with their objectives. Sustainable investment, also referred to as Socially Responsible Investment (SRI), seeks to attain financial returns while simultaneously generating positive social or environmental impacts. It encompasses various approaches, including avoiding investments in harmful activities, actively selecting companies with strong sustainability performance, engaging with companies to drive sustainability improvements, and impact investing that focuses on specific social or environmental impacts. Sustainable investing prioritizes choosing companies that meet ESG criteria to achieve positive social and environmental outcomes while also generating financial returns. Investors should consider their financial goals, risk tolerance, and investment timeframes when selecting an investment strategy. It’s important to regularly review and adjust strategies to align with changing market conditions and financial objectives^30–37^. Assessing risk tolerance is an essential initial step in choosing an appropriate investment strategy, as it helps investors understand their capacity and willingness to tolerate potential investment losses. By understanding their risk tolerance, investors can select an investment strategy that aligns with their financial objectives and effectively manages investment risks.

**Figure 3:**
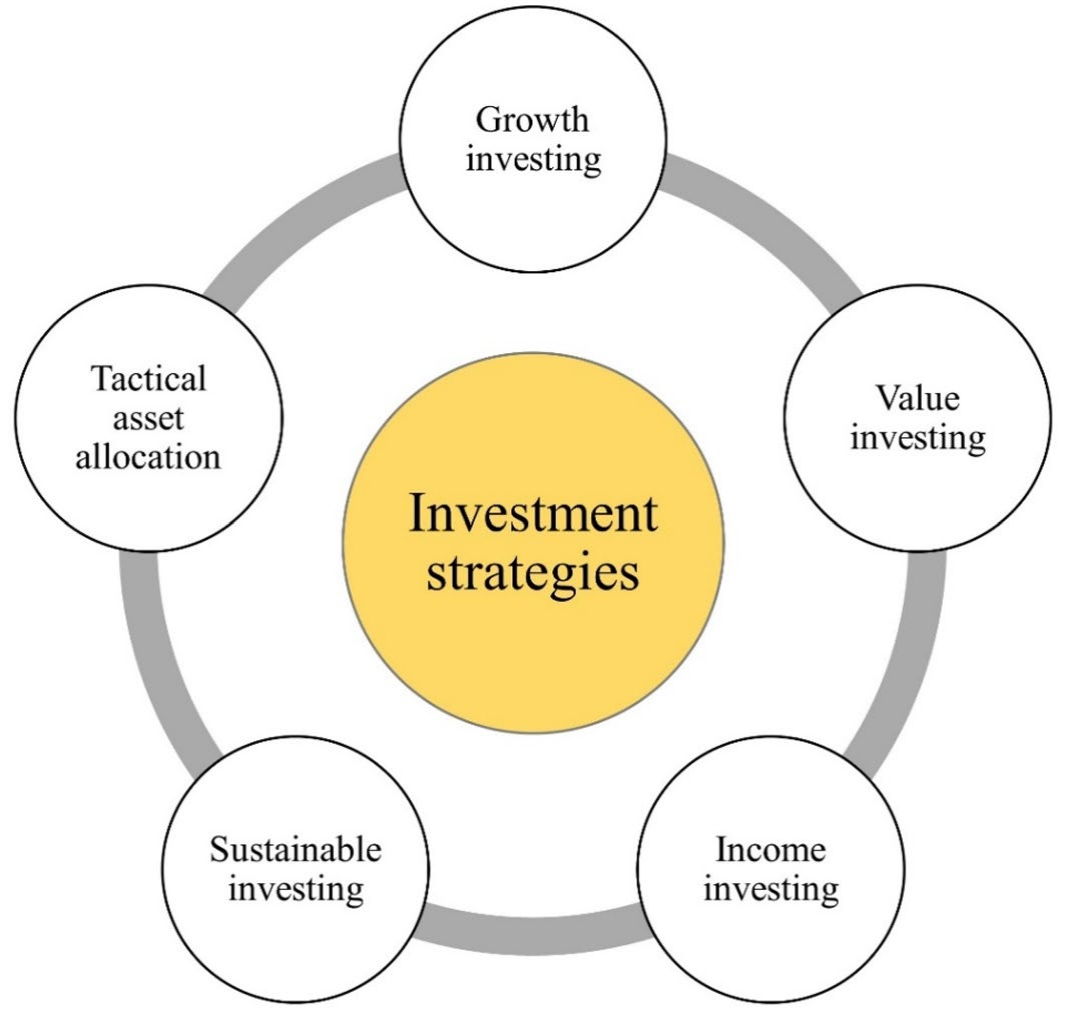
Various investment strategies including growth investing, value investing, income investing, index investing, sustainable investing, and tactical asset allocation.

Figure 3 shows the investment strategies, illustrating their distinctive characteristics, objectives, and approaches, emphasizing the importance of aligning investment decisions with individual goals, risk tolerance, and market conditions. Investment strategies play a crucial role in guiding investment decisions and managing portfolios, aiming to achieve specific objectives while aligning with investors' risk tolerance, time horizon, and financial goals. Growth investing focuses on high-growth potential companies, aiming for capital appreciation through investments in stocks of companies expected to experience above-average growth in earnings and revenue^40–42^.

Value investing involves identifying undervalued stocks, with the expectation that their intrinsic value will be recognized over time, focusing on companies trading below their intrinsic value due to market inefficiencies or negative sentiment. Income investing aims to generate a consistent stream of income by investing in assets providing regular cash flows, prioritizing current income over capital appreciation through investments such as dividend-paying stocks, bonds, REITs, and other income-generating assets. Index investing replicates the performance of a specific market index through a diversified portfolio mirroring the index’s composition, characterized by lower costs, broad market exposure, and a long-term investment approach. Sustainable investing incorporates ESG factors into investment decisions, seeking financial returns while considering societal and environmental impact, implemented through various approaches like negative screening, positive screening, and impact investing. Tactical asset allocation adjusts portfolio asset allocation based on short-term market conditions and opportunities, deviating from predetermined allocations to capitalize on market inefficiencies and short-term trends, requiring active management and regular market monitoring^41–44^.

### The importance of sustainability criteria and choosing the right investment strategy

Investors are increasingly focused on sustainability criteria to align their investments with their values and drive positive social and environmental impacts. Companies are also acknowledging the importance of sustainability and improving their performance to attract investment and maintain their societal acceptance^43–45,47^. Sustainability criteria help investors evaluate companies’ sustainability performance and make value-aligned investment decisions. Investment strategies, such as growth investing, value investing, income investing, index investing, sustainable investing, and tactical asset allocation, guide investors’ decisions based on their financial goals, risk tolerance, and investment timeframes. Sustainable investing places particular emphasis on companies that meet ESG criteria, aiming for positive social and environmental outcomes alongside financial returns^48–50^. This approach enables investors to support sustainability and contribute to a better future. Selecting an investment strategy requires aligning it with financial goals, risk tolerance, and investment timeframe to ensure it meets individual needs and objectives. Regularly reviewing and adjusting the strategy based on evolving market conditions and financial goals is also important. Additionally, assessing risk tolerance is a vital initial step in choosing a suitable investment strategy, as it helps investors understand their capacity and willingness to accept potential investment losses. By understanding their risk tolerance, investors can select an investment strategy that matches their financial objectives and effectively manages investment risks. The sustainability criteria play a significant role in guiding investors toward sustainable investment decisions. By considering these criteria and choosing the right investment strategy, investors can align their investments with their values, promote positive social and environmental impacts, and work towards a more sustainable future.

Various scholarly articles have covered a wide range of topics, including achieving consensus on food safety through the utilization of blockchain technology^53^, implementing adaptive control techniques for teleoperation systems operating under uncertain dynamics^54^, monitoring changes in house vacancy dynamics using remote sensing images^55^, examining the impact of low-carbon strategies on the digital transformation of manufacturing enterprises^56^, employing multiscale feature extraction and fusion methods to enhance visual question answering systems^57^. An exploring quantum detectable Byzantine agreement as a means of ensuring trustworthy data management in blockchain systems^58^, investigating the application of H_∞_ consensus algorithms in multiagent-based supply chain systems^59^, analyzing the effects of digital technology promotion on the disruption of technology innovation efficiency^60^, studying the relationship between financial inclusion and energy productivity^61^, proposing public verifiable and forward-privacy encrypted search mechanisms using blockchain technology^62^. Also, measuring and decomposing innovation inequality^63^, evaluating changes in corporate social responsibility efficiency in the Chinese food industry during the COVID-19 pandemic^64^, examining optimization measures for green energy projects and their impact on CO_2_ emissions^65^, investigating avoidable hospitalizations related to diabetes and primary healthcare resource allocation in China^66^. Several suggesting strategies for reducing the urban carbon footprint in China based on the Tapio Decoupling Principle^67^, developing a many-objective optimization model for the industrial IoTs based on private blockchain^68^, analyzing the concept of partial centralization in durable-good supply chains^69^, integrating traditional Chinese medicine ideology into modern compensation management practices^70^, and investigating the formation and operation mechanisms of self-organization among delivery riders^71^.

Risk tolerance accounts for an investor’s willingness and ability to tolerate fluctuations in investment value, taking into consideration factors such as risk preferences, time horizon, financial situation, and investment knowledge. The investment horizon reflects the intended duration of investment holdings, ranging from short-term to medium-term or long-term^32–35^. The investment horizon influences strategy selection, as longer-term horizons may allow for more exposure to growth-oriented investments, while shorter-term horizons may require a more cautious approach to mitigate short-term market fluctuations. Regular review and adjustment based on market conditions and goals are essential for successful investment strategies. Financial markets are dynamic and influenced by various factors, necessitating ongoing monitoring and adaptation^36–40^. Regularly reviewing investment portfolios enables investors to assess performance, make necessary adjustments, and ensure alignment with financial goals and risk tolerance. This process may involve rebalancing portfolios, adjusting asset allocation, or making strategic investment decisions based on changing market conditions^41–45,47,48^.

**Figure 4:**
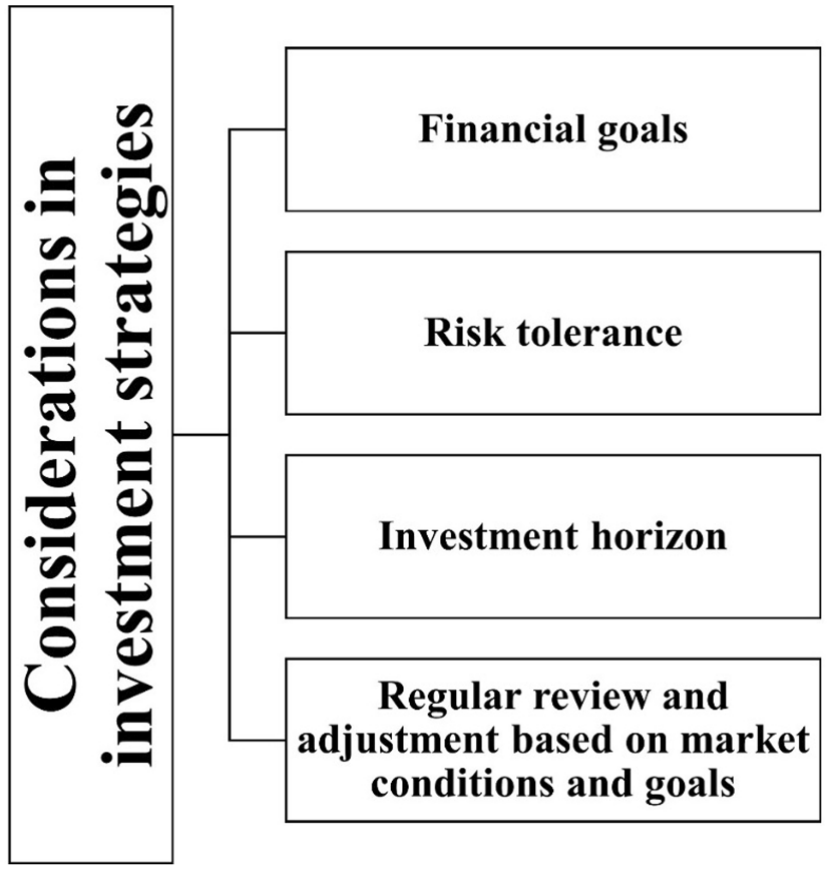
Considerations in investment strategies, financial goals, risk tolerance, investment horizon, and regular review and adjustment based on market conditions and goals.

Figure 4 represents the key considerations in investment strategies, including financial goals, risk tolerance, investment horizon, and the importance of regular review and adjustment based on market conditions and goals. These factors provide essential guidance for investors, ensuring that their investment approach aligns with their individual circumstances and objectives^41–44^. Financial goals serve as the foundation for investment strategies, providing a clear direction for selecting appropriate approaches that are most likely to achieve desired outcomes such as capital appreciation, income generation, or wealth preservation.

Figure 4 shows the considerations, emphasizing their interconnectedness and guiding investors towards a comprehensive approach to investment decision-making. By highlighting the importance of aligning investment strategies with financial goals, risk tolerance, investment horizon, and the need for regular review and adjustment, also serves as a reminder that successful investment strategies require continuous monitoring, adaptation, and alignment with individual circumstances and market dynamics^48–52^. When selecting an investment strategy based on risk tolerance, it is crucial to avoid common mistakes, such as failing to regularly rebalance your portfolio. To choose an investment strategy that aligns with your risk tolerance, carefully consider factors such as your investment time horizon, financial goals, and personal circumstances.

### Future work and suggestions

This research article suggest to explore the implications of sustainable development, addressing environmental degradation and mitigation strategies in the power generation, cement manufacturing, automotive, and food and beverage industries, while also investigating the potential role of emerging technologies such as blockchain and AI in promoting sustainability within supply chain management, discussing their ability to enhance transparency, traceability, and efficiency, and examining relevant research, pilot projects, and industry applications to provide a comprehensive analysis of the environmental, social, and economic benefits of adopting sustainable practices and the potential for mitigating environmental impacts and improving overall environmental performance in these sectors. Expand on the concept of sustainable development and its relevance to the sectors discussed in your article. Discuss the potential environmental, social, and economic benefits of adopting sustainable practices in power generation, cement manufacturing, automotive, and food and beverage industries. Consider incorporating relevant theories or frameworks related to sustainable development, such as the triple bottom line or circular economy principles, to provide a comprehensive analysis.

## Conclusion

In conclusion, the integration of innovative digital technologies provides significant opportunities for optimizing production systems and economic activity while focusing on sustainability objectives for the betterment of society and the environment. These technologies hold the potential to mitigate environmental externalities by addressing information imbalances within global supply chains. However, it is crucial to prioritize inclusive governance that emphasizes democratic participation in order to mitigate any unintended negative consequences, particularly for vulnerable communities. By ensuring inclusive decision-making processes, we can maximize the positive impact of these technologies while minimizing potential harm. Collaborative policymaking that incorporates diverse perspectives provides a prudent approach to guide responsible innovation that benefits present and future generations. The establishment of multi-stakeholder cooperatives that incorporate local perspectives represents constructive arenas for scaling effective solutions and ensuring widespread cooperation in navigating the rapidly changing landscape. With careful stewardship and a focus on well-being as the primary directive, these emerging tools can help accelerate the transition to post-carbon economies while fostering shared prosperity within the boundaries of our planet. The combination of AI and blockchain enhances efficiency, transparency, and traceability throughout the supply chain, enabling better resource management and waste reduction. AI algorithms optimize production processes, reduce energy consumption, and enhance decision-making, leading to more sustainable practices. Blockchain technology, on the other hand, provides secure and immutable data storage, fostering transparency and trust among stakeholders. Leveraging the power of AI and blockchain enables organizations to achieve significant advancements in sustainability by promoting responsible resource management, minimizing environmental footprints, and fostering sustainable development practices.

## Data Availability

All data generated or analyzed during this study are included in this published article.
